# The Socialization of Perceived Discrimination in Ethnic Minority Groups

**DOI:** 10.1037/pspi0000426

**Published:** 2023-06-19

**Authors:** Chloe Bracegirdle, Nils Karl Reimer, Danny Osborne, Chris G. Sibley, Ralf Wölfer, Nikhil Kumar Sengupta

**Affiliations:** 1 Nuffield College, University of Oxford; 2 Department of Psychological and Brain Sciences, University of California, Santa Barbara; 3 School of Psychology, University of Auckland; 4 Hochschule des Bundes für öffentliche Verwaltung; 5 School of Psychology, University of Kent

**Keywords:** perceived discrimination, ingroup contact, intergroup contact, socialization, social network analysis

## Abstract

Contact with members of one’s own group (ingroup) and other groups (outgroups) shapes individuals’ beliefs about the world, including perceptions of discrimination against one’s ingroup. Research to date indicates that, among members of disadvantaged groups, contact with an advantaged outgroup is associated with less perceived discrimination, while contact with the disadvantaged ingroup is associated with more perceived discrimination. Past studies, however, considered ingroup and outgroup contact in isolation and overlooked the various processes that could explain these associations. We addressed these issues by examining whether disadvantaged-group members’ perceptions of discrimination are shaped by how much contact they have with ingroup and outgroup members (*contact effects*) or by those ingroup and outgroup members’ perceptions of discrimination (*socialization effects*) while controlling for their tendency to affiliate with similar others (*selection effects*). Three studies (total *N* = 5,866 ethnic minority group members) assessed participants’ positive contact, friendships, and perceived discrimination and applied longitudinal and social network analyses to separate and simultaneously test contact, socialization, and selection processes. In contrast to previous studies, we found no evidence that contact with members of the advantaged outgroup precedes perceived discrimination. Instead, we found that friendships with members of the disadvantaged ingroup longitudinally predict perceived discrimination through the process of socialization—disadvantaged-group members’ perceptions of discrimination became more similar to their ingroup friends’ perceptions of discrimination over time. We conclude that perceptions of discrimination should be partly understood as a socialized belief about a shared reality.

Discrimination in education, employment, housing, policing, and other domains is ubiquitous for members of disadvantaged groups. *Perceiving* discrimination, however, requires a subjective judgment that involves attributing negative events or outcomes to systemic unfairness against one’s group (Major et al., 2002). Perceived discrimination thus partly reflects beliefs about the workings of the prevailing sociopolitical system (Bahamondes, Sibley, et al., 2021), which are rooted in ideologies that often legitimize the existing social order as fair and just (Jost, 2020; Major & Kaiser, 2017; Sidanius & Pratto, 1999). Ideologies, in turn, result from social, motivational, and other processes that can prevent people’s perceptions from accurately reflecting reality (Dawtry et al., 2019; Kay et al., 2009). Indeed, research shows that people, including members of disadvantaged groups, often underestimate the degree of discrimination in society (Hauser & Norton, 2017; Kraus et al., 2019; Sengupta, Osborne, et al., 2015).

Subjective though it may be, perceived discrimination may have both individual and group-level consequences for members of disadvantaged groups. Research and theorizing on relative deprivation emphasize that subjective perceptions of disadvantage, rather than the objective reality, motivate the disadvantaged to challenge social injustice (Smith et al., 2012). Related work similarly shows that perceiving discrimination is often a prerequisite for gaining political power and greater equality (van Zomeren et al., 2008; Wright, 2003). At the same time, perceiving discrimination can negatively affect the physical and mental health of disadvantaged-group members (Pascoe & Smart Richman, 2009; Schmitt et al., 2014), providing a motivation to overlook discrimination to protect one’s well-being (Osborne & Sibley, 2013; Sengupta et al., 2017). Perceiving discrimination may thus come with costs to the individual but has potential benefits for the group (e.g., gaining political power) and society (e.g., reducing inequality). It is, therefore, of critical importance to understand how perceptions of discrimination are formed and developed over time.

We extend research on the subjective and ideological nature of perceived discrimination by proposing that it should be subject to the processes of social contact and social influence. Prior research indicates that social relationships with members of the disadvantaged ingroup and advantaged outgroup might have important, but opposing, effects on disadvantaged-group members’ perceptions of discrimination. Specifically, ingroup contact is associated with higher perceived discrimination (Levin et al., 2006; Sengupta, Milojev, et al., 2015), while outgroup contact is associated with lower perceived discrimination (Dixon et al., 2010; Tropp et al., 2012). Yet, it is unclear whether these associations reflect *contact effects* (time spent with ingroup or outgroup members), *socialization effects* (individuals taking on the perceptions of those ingroup or outgroup members), or *selection effects* (individuals forming relations with those who already share their views). In the present research, we use longitudinal and social network analyses to separate these distinct processes, simultaneously examine their effects, and thus explore how social relationships, with ingroup and outgroup members, might shape perceived discrimination.

In so doing, we extend past research in three directions. First, we add to the few published studies on perceived discrimination that measure both ingroup and outgroup contact, allowing us to estimate the distinct effects of both types of social relations. Second, we present the first longitudinal study of these effects, which allows us to examine the direction of the hypothesized relationships while disentangling within-person change from between-person stability (see Hamaker et al., 2015; Osborne & Little, in press). Finally, by drawing on recent advances in longitudinal social network analysis to simultaneously model the effects of contact, socialization, and selection, we shed light on the processes by which social relationships may shape perceptions of discrimination.

## Contact and Perceived Discrimination

To paint a complete picture of how social relationships influence disadvantaged-group members’ perceptions of discrimination, we must consider contact with members of both the disadvantaged ingroup and the advantaged outgroup. Contact can take many forms, ranging from short-term interactions to long-term relationships such as friendships (MacInnis & Page-Gould, 2015), and can be positive, negative, or relatively neutral in valence (Schäfer et al., 2021). We focus specifically on direct, qualitatively positive contact and friendship, as these forms of contact have been found most consistently associated with perceived discrimination (Reimer & Sengupta, 2023).

Guided by intergroup contact theory (Allport, 1954; Brown & Hewstone, 2005), most research to date has focused on contact with outgroup members and its well-documented promise for improving outgroup attitudes (Pettigrew & Tropp, 2006; Tropp & Pettigrew, 2005). While outgroup contact may improve outgroup attitudes, outgroup contact is also associated with other constructs including reduced ingroup identification, collective action, and support for social change in disadvantaged groups (Cakal et al., 2011; Hässler et al., 2020; Tausch et al., 2015). Outgroup contact is accordingly considered to have both positive and negative consequences for various aspects of intergroup relations.

With regard to perceived discrimination, Dixon et al. (2010) proposed that, if disadvantaged-group members’ interactions with advantaged-group members were generally positive, outgroup contact experiences would reduce perceived personal discrimination and, in turn, perceived group discrimination. Additionally, if outgroup contact improved attitudes toward the advantaged, it could render discrimination less plausible. Positive attitudes toward the advantaged outgroup may therefore be linked to perceiving less discrimination from the advantaged outgroup. Consistent with this expectation, meta-analytic evidence indicates an association, albeit weak, between positive outgroup attitudes and lower perceived discrimination among members of disadvantaged groups (*r* = −.10, [−.02, −.18]; Reimer & Sengupta, 2023). Meta-analytic evidence also indicates an association between perceived discrimination and collective action (*r* = .31, [.23, .38]; Reimer & Sengupta, 2023; *r* = .35, [.30, .39]; van Zomeren et al., 2008), such that disadvantaged-group members who perceive greater discrimination are more likely to engage in collective action. Meta-analytic evidence thus suggests that perceived discrimination, outgroup attitudes, and collective action are related, but distinct, constructs.

Prior research has tested the hypothesis that outgroup contact reduces perceived discrimination in disadvantaged groups. A recent meta-analysis found that, across 203,637 participants in 86 studies, contact with the advantaged was, on average, associated with less perceived discrimination among the disadvantaged (Reimer & Sengupta, 2023). This association, however, was small (between −.10 < *r* < −.04) and variable (31% of studies found a *positive* association). Furthermore, the available evidence consisted predominantly of cross-sectional survey studies (e.g., Dixon et al., 2010), which cannot establish the direction of the association: Outgroup contact could reduce perceived discrimination, or perceiving greater discrimination could lead disadvantaged-group members to engage in less outgroup contact (Tropp et al., 2012). Longitudinal, experimental, and intervention studies can test the direction of the association. Yet, the available evidence includes few longitudinal studies (Koschate et al., 2012; Reimer et al., 2017; Tropp et al., 2012), all of which conflated within-person change and between-person stability (Hamaker et al., 2015), and only one intervention study (Reimer et al., 2021) and one experimental study (Saguy et al., 2009), both of which omitted a control group. Of these longitudinal, experimental, and intervention studies, only two (Saguy et al., 2009; Tropp et al., 2012) found that outgroup contact predicted less perceived discrimination. Therefore, the available evidence generally suggests outgroup contact is associated with less perceived discrimination in disadvantaged groups, but evidence for causality is scarce.

Fewer studies have examined whether ingroup contact affects perceived discrimination. Levin et al. (2006) proposed that, for members of disadvantaged groups, ingroup peers provide an important reference group and that, to the extent that perceiving discrimination is normative within the group, ingroup contact can reinforce perceptions of discrimination. Consistent with this argument, the researchers found that, among Asian, Latino, and African American students, having more ingroup friends in their first year of college predicted perceiving more discrimination against their group in their second and third years. Similarly, Sengupta, Milojev, et al. (2015) drew on evidence for the relational nature of political attitudes (see Jost et al., 2008, for a review) to propose that ingroup contact shapes ideological beliefs by fostering shared understandings of the social world. In a large, nationally representative sample of Māori (i.e., the indigenous peoples and the largest ethnic minority group of New Zealand), ingroup contact correlated negatively with the belief that all ethnic groups are treated fairly in New Zealand. Together, these studies provide tentative evidence that ingroup contact might increase perceived discrimination in disadvantaged groups.

Researchers have argued that people have a limited capacity for social relationships and showed that, in line with this argument, individuals who have more ingroup friendships tend to have fewer outgroup friendships and vice versa (Pfister et al., 2020). Accordingly, studies need to consider both ingroup and outgroup contact in order to avoid confounding the two. While several studies have examined the joint effects of ingroup and outgroup contact on outgroup attitudes (e.g., Munniksma et al., 2015), only one study to date has examined the joint effects of ingroup and outgroup contact on perceived discrimination in disadvantaged groups. Sengupta and Sibley (2013) found that, among Māori in New Zealand, ingroup and outgroup contact were respectively associated with rejecting and endorsing meritocracy, which implies that group differences in resources are the result of individual merit rather than group discrimination.^1^ Based on the evidence reviewed, we expect ingroup and outgroup contact to have opposite effects, with the former increasing perceptions of discrimination and the latter decreasing perceptions of discrimination.

## Socialization and Perceived Discrimination

The research discussed thus far concerns the effects of having more or less contact with ingroup and outgroup members on disadvantaged-group members’ perceptions of discrimination. There are, however, other ways in which contact, especially friendships, may shape perceived discrimination. Individuals may adjust their attitudes, beliefs, and behaviors to become more similar to those of valued groups and individuals (Hardin & Higgins, 1996; Turner, 1991). Kandel (1978) termed this process *socialization*, defined as the increase in similarity between friends over time through mutual influence. Through the process of socialization, disadvantaged-group members’ perceptions of discrimination might be influenced not merely by how many ingroup and outgroup friends they have, but also by their friends’ perceptions of discrimination. Accordingly, friends’ perceptions of discrimination should become more similar over time.

Past research suggests that socialization may shape sociopolitical beliefs, attitudes, and ideologies. Oosterhoff et al. (2022) found that friends tended to share similar levels of patriotism, right-wing authoritarianism, and anti-immigration attitudes. Similarly, Stangor and Leary (2006) identified similarities between friends in social dominance orientation, right-wing authoritarianism, and prejudice toward various outgroups. Such cross-sectional studies provide evidence of shared sociopolitical attitudes and ideologies between friends but cannot determine whether the similarity results from friends influencing each other over time (i.e., socialization) or individuals choosing to befriend similar others (i.e., selection).

Critically, both processes are plausible. Individuals can be influenced by their friends and also select as friends those who are similar with regard to various sociodemographic, behavioral, and intrapersonal characteristics (McPherson et al., 2001). Longitudinal social network analysis allows one to separate and simultaneously examine socialization and selection processes. While this method remains underutilized in social psychology (Wölfer & Hewstone, 2017), it has been applied to study the socialization of attitudes toward outgroups. Longitudinal social network studies have found that friends inﬂuence each other’s attitudes toward outgroups over time while controlling for individuals’ tendencies to select friends who already hold similar attitudes (Bracegirdle et al., 2022; van Zalk et al., 2013; Zingora et al., 2020). These findings suggest that socialization might also shape other sociopolitical attitudes, beliefs, and ideologies, including perceived discrimination.

Several researchers have proposed that socialization explains why ingroup contact might *increase* perceived discrimination (Levin et al., 2006; Sengupta, Milojev, et al., 2015; Sengupta & Sibley, 2013). During ingroup contact, disadvantaged-group members might exert reciprocal social influence on each other, resulting in their perceptions of discrimination becoming more similar and shifting toward the shared norm within the disadvantaged ingroup. This may not only increase the alignment of perceptions but also foster polarization in the direction of the group norm (Haslam et al., 1999). Reflecting disadvantaged-group members’ collective experiences and shared opposition to group-based hierarchies (Lee et al., 2011), this process could explain why ingroup contact increases perceived discrimination. During ingroup contact, disadvantaged-group members might further discuss personal experiences of discrimination, which, in turn, could make group discrimination seem more prevalent.

In theory, socialization could also explain the effect of outgroup contact. Due to either a lack of direct experience or ideological reasons, advantaged-group members often perceive less discrimination against the disadvantaged outgroup than do disadvantaged-group members (Norton & Sommers, 2011). Accordingly, contact with the advantaged could, through social influence, reduce perceived discrimination among the disadvantaged. Socialization is thus a plausible explanation for the opposite effects of ingroup and outgroup contact on perceived discrimination.

Socialization may, however, be more likely to occur through relationships with ingroup members than with outgroup members. Theoretical work contends that individuals conform to the norms of groups with which they identify (Sherif & Sherif, 1953; Turner et al., 1987) and adjust their attitudes in accordance with similar others to achieve uniformity within a social group (Festinger, 1954). Furthermore, disadvantaged-group members may be influenced only by ingroup members’ perceptions of discrimination because outgroup members’ perceptions could be considered biased, untrue, or based on indirect experience. Bracegirdle et al. (2022) conducted the first social network study to distinguish ingroup and outgroup socialization and found that ingroup, not outgroup, friends’ attitudes influenced individuals’ attitudes toward ethnic outgroups. Consistent with these findings, socialization through ingroup friends, rather than outgroup friends, may shape perceived discrimination in disadvantaged groups. Research to date could not test whether socialization shapes perceived discrimination because it measured only how much contact with ingroup and outgroup members someone has—and not what those ingroup and outgroup members’ perceptions of discrimination are. In the present research, we simultaneously examine both contact and socialization, in order to consider the multiple ways in which social relationships may inﬂuence perceived discrimination.

## The Present Research

The reviewed research gives reason to expect that perceived discrimination may be shaped through the processes of contact and socialization. On the basis of this literature, we propose three hypotheses specifying how social relationships with ingroup and outgroup members might influence perceptions of discrimination among disadvantaged-group members. First, we hypothesize that greater outgroup contact will predict decreased perceived discrimination (termed an “outgroup contact” effect). Second, we hypothesize that greater ingroup contact will predict increased perceived discrimination (termed an “ingroup contact” effect). Third, we hypothesize that ingroup friends’ perceptions of discrimination will influence individuals’ perceptions of discrimination, such that their perceptions will become more similar over time (termed an “ingroup socialization” effect). Finally, we explore the extent to which outgroup friends’ perceptions of discrimination influence individuals’ perceptions of discrimination (termed an “outgroup socialization” effect). As no study to date has investigated the socialization of perceived discrimination, we seek to determine whether socialization occurs only through ingroup friends, or also through outgroup friends.

We combine different state-of-the-art methods to test our hypotheses across three studies.^2^ The first study examines the longitudinal associations between ingroup and outgroup contact and perceived discrimination among ethnic minority groups in New Zealand. This study does not investigate socialization but rather seeks to determine whether the associations identified in prior research will replicate when separating within-person change and between-person stability (Hamaker et al., 2015). The second study uses Bayesian multilevel network autocorrelation modeling to examine ingroup and outgroup socialization, in addition to ingroup and outgroup contact, among ethnic minority students in the United Kingdom. This study investigates the associations between individuals’ perceptions of discrimination, their numbers of ingroup and outgroup friends, and their friends’ perceptions of discrimination. The third study uses longitudinal social network analysis to explore the direction of the associations tested in Study 2 among a sample of Asian British students. This study examines whether friends have similar perceptions of discrimination because individuals are influenced by their friends’ perceptions (i.e., socialization) or because individuals choose friends with similar perceptions (i.e., selection). The intergroup contexts and ethnic groups examined therefore differ across the three studies.

We consider two forms of contact, consisting of direct positive contact in Study 1 and friendship in Studies 2 and 3. Research suggests that direct positive contact and friendship are more consistently associated with perceived discrimination than indirect, neutral, and negative contact (Reimer & Sengupta, 2023). Friendship may be especially important for shaping individuals’ perceptions of discrimination because friendship occurs over an extended period of time and entails active engagement, self-disclosure, empathy, intimacy, and trust (MacInnis & Page-Gould, 2015; Swart et al., 2011). Furthermore, although individuals may be influenced by unfamiliar others in short interactions (Blanchard et al., 1991), socialization may be more likely to occur through long-term intimate interactions with those who are liked, especially one’s friends (Paluck, 2011; Sinclair et al., 2005). In the present research, we investigate the extent to which both friendship and generic positive contact shape perceived discrimination.

## Study 1

Prior research testing the effects of ingroup and outgroup contact on perceived discrimination has relied almost exclusively on cross-sectional surveys (e.g., Dixon et al., 2010; see Reimer & Sengupta, 2023). For this reason, it is unclear whether contact leads to a change in perceived discrimination, perceived discrimination leads to a change in contact, both, or neither. Studies that assess contact and perceived discrimination over time can provide evidence for the temporal order of effects by identifying whether changes in ingroup or outgroup contact *precede* changes in perceived discrimination or vice versa. Temporal precedence is a necessary (but not sufficient) condition for causal inference.

However, the few longitudinal studies in this literature have used cross-lagged panel modeling (CLPM; e.g., Tropp et al., 2012), which produces biased estimates because CLPMs fail to separate time-invariant, between-person stability from within-person change (Hamaker et al., 2015; Orth et al., 2021; Osborne & Little, in press). Applied to contact research, between-person stability reflects the degree to which those who report high (vs. low) levels of contact *across all time points* also report high (vs. low) perceived discrimination across all time points. Between-person stability can be considered a confound when estimating causal effects (Granger, 1980) and may indicate that unobserved variables produce an artificial correlation between stable individual differences in contact and perceived discrimination (i.e., possible third-variable effects) or may reflect prior changes in a subsequently stable environment. Most importantly, between-person stability tells us little about the extent to which a change in contact leads to a change in perceived discrimination. In contrast, identifying within-person change would mean that an increase or decrease from one’s average level of contact at a given time point precedes an increase or decrease from one’s average level of perceived discrimination *at the following time point*. Thus, after adjusting for between-person stability, within-person change demonstrates the temporal precedence of contact relative to perceived discrimination, which provides stronger evidence of an effect of contact on perceived discrimination.

**Figure 1 fig1:**
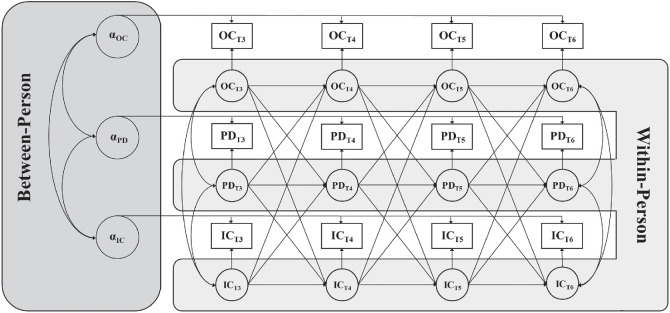
Random-Intercept Cross-Lagged Panel Model *Note*. RI-CLPM = random-intercept cross-lagged panel model; OC = outgroup contact; PD = perceived discrimination; IC = ingroup contact. Conceptual RI-CLPM of the associations between OC, IC, and PD. Due to space constraints, the contemporaneous residual covariances were excluded from the figure.

Hamaker et al. (2015) developed an extension to the CLPM, termed the random-intercept cross-lagged panel model (RI-CLPM; see Figure 1), to separately estimate between-person stability and within-person change. Although there are alternative methods for separating between-person and within-person effects, RI-CLPMs are the most likely to converge (Orth et al., 2021) and do not require intensive longitudinal data (e.g., over 30 assessments). Thus, RI-CLPMs are quickly becoming the method of choice amongst longitudinal researchers interested in within-person effects (Friehs et al., 2023; O’Donnell et al., 2021; Osborne & Little, in press). Here, we apply this model to test, for the first time, whether levels of ingroup and outgroup contact predict within-person change in perceived discrimination over time. This method also allows us to investigate potential reciprocal effects, in which perceived discrimination predicts within-person change in contact over time.[Fig fig1]

### Method

#### Participants and Procedure

Study 1 draws on data from a large-scale national longitudinal study in New Zealand: The New Zealand Attitudes and Values Study (NZAVS). New Zealanders of European descent (hereafter, Europeans) represent the advantaged ethnic group. The indigenous Māori, as well as later immigrant groups from the Pacific Islands and Asia, represent disadvantaged ethnic groups. Ethnic minority groups in New Zealand face longstanding deficits relative to Europeans across a range of socioeconomic indicators including income, unemployment, life expectancy, and life satisfaction (Ministry of Social Development, 2016). Thus, our analyses focus on the contact each ethnic minority group—Māori, Pacific Islanders, and Asians—has with their own group (ingroup contact) and with Europeans (outgroup contact) and whether this affects their perceptions of discrimination against their own ingroup.

The NZAVS began in 2009 (i.e., Time 1). Invitations to participate in Time 1 were sent to 40,500 people randomly selected from the electoral roll, 6,518 (16.6%) of whom returned completed surveys. In 2010, Time 2 surveys were sent to all participants who responded at Time 1. This survey was completed by 4,423 (67.9%) participants from the Time 1 sample. From Time 3 to Time 5 (2011–2013), in addition to sending out surveys to existing participants, booster sampling was conducted to increase the overall sample size (see Sibley, 2018). Thus, Time 3 (2011) contained responses from 6,844 participants, Time 4 (2012) had 12,182 participants, Time 5 (2013) had 18,264 participants, and Time 6 (2014) had 15,822 participants.

The measures required for the present research (i.e., contact and perceived discrimination) were only included in the NZAVS survey at Times 3, 4, 5, and 6. Accordingly, data for the present study were drawn from the only four consecutive waves of the NZAVS that contained all the items required for our model: Time 3 (2011) to Time 6 (2014). This yielded a total sample size of 4,325 ethnic minority participants (2,929 Māori, 558 Pacific Islanders, and 839 Asians) who provided partial or complete responses to the variables of interest and who responded to at least one of the four surveys (*M*_age_ = 47.43, *SD* = 13.73; 65.87% women). Of these participants, 842 (19%) completed all four surveys, 1,271 (29%) completed three surveys, 1,920 (44%) completed two surveys, and 292 (7%) completed only one survey. Across surveys, 81% (Time 4), 84% (Time 5), and 84% (Time 6) of participants who participated in the preceding wave (Times 3–5) participated in the subsequent wave (Times 4–6).^3^ Descriptive statistics for all variables included in the model are presented in online Appendix A.^4^ No a priori power analysis was conducted. However, due to our large sample size and long study duration, we had adequate statistical power to detect even very small longitudinal effects.

#### Measures

##### Ingroup and Outgroup Contact

We assessed contact with a widely used measure (see Barlow et al., 2012) that asks: “How frequently do you have positive/good interactions with [New Zealand Europeans/Māori/Pacific Islanders/Asians]?” (1 = *not frequently at all*, 7 = *very frequently*). Using these items, we constructed a measure of outgroup contact that represented the frequency of contact Māori, Pacific Island, and Asian participants had with Europeans and a measure of ingroup contact that represented the frequency of contact Māori, Pacific Island, and Asian participants had with their own respective ethnic ingroup.

##### Perceived Group Discrimination

Consistent with prior theory and research on the construct of perceived discrimination, we assessed perceived discrimination in terms of people’s perceptions that their ingroup is discriminated against in society (see Branscombe et al., 1999). We used a single-item measure that asked, “Do you think people from your ethnic group are discriminated against in New Zealand?” (1 = *definitely no*, 7 = *definitely yes*).

#### Analysis Strategy

Following the RI-CLPM procedure outlined by Hamaker et al. (2015), each observed variable was modeled as a function of two latent variables. One latent variable was the time-invariant random intercept, which was modeled as loading equally on each congeneric indicator across time (e.g., the random intercept of ingroup contact loaded equally on ingroup contact at Times 3, 4, 5, and 6). The second latent variable loaded only on its corresponding indicator at each individual wave, thus representing a time-specific departure from the average response over time. The error variances of the observed variables were constrained to zero meaning that all variation in the observed scores was explained by the within-person and between-person factor structure.

The random intercepts of ingroup contact, outgroup contact, and perceived discrimination were then correlated with each other to estimate between-person effects. These correlations indicate the degree to which stable individual differences in contact are associated with stable individual differences in perceived discrimination (i.e., trait-like stability). Having thus accounted for between-person effects, the time-specific latent variables for ingroup contact, outgroup contact, and perceived discrimination were regressed on each other in the same manner as a traditional CLPM, yielding autoregressive and cross-lagged coefficients (e.g., perceived discrimination at Time 4 regressed on ingroup and outgroup contact at Time 3; ingroup and outgroup contact at Time 4 regressed, in turn, on perceived discrimination at Time 3). These coefficients represent within-person change over time—the degree to which an individual’s deviation from their expected score (i.e., their own mean across waves) on the predictor variable at a given time point explains their deviation from their expected score on the outcome variable at the following time point.

Finally, we estimated the model as a stationary process by constraining the corresponding autoregressive and cross-lagged effects to equality across time. This approach maximizes power and represents an assumption of a continuous process with an unknown starting point (McArdle, 2009). We estimated the model using Mplus 8.4 (Muthén & Muthén, 2015). The outcome variables, which we analyzed as continuous variables, had 7-point response scales. Analyzing ordinal data as if they were metric data can distort estimates of effect sizes and inflate rates of false-positive and false-negative findings (Liddell & Kruschke, 2018). But, as the RI-CLPM has not been implemented or described for ordinal variables, we nonetheless proceeded to model all variables as continuous variables. The materials and analysis code for Study 1 are provided online, and the data are available upon request (see online Appendix D).

### Results and Discussion

#### Model Fit

Because a traditional CLPM reflects a constrained version of an RI-CLPM (i.e., the CLPM is a model in which the variances and covariances of the random intercepts are constrained to zero), it is possible to empirically evaluate whether the RI-CLPM provides a better fit to these data than a traditional CLPM. To test this formally, we compared the fit of our model with a CLPM. Results showed that the RI-CLPM of contact and perceived discrimination fit these data well, χ^2^(39) = 56.361; comparative fit index [CFI] = .999; root-mean-square error of approximation [RMSEA] = .009, 95% CI [<.001, .015]; standardized root-mean-square residual [SRMR] = .020, and significantly better than the CLPM, χ^2^(45) = 1023.721; CFI = .897; RMSEA = .071, 95% CI [.067, .075]; SRMR = .083; Δχ^2^(6) = 967.36; *p* < .001. These results demonstrate the importance of separating the between-person and within-person effects in our data.

#### Between-Person Correlations

Results from the RI-CLPM showed that the random intercept for ingroup contact was positively correlated with the random intercept for perceived discrimination (*r* = .30, *SE* = .04, *p* < .001; 99% CI [.21, .37]), whereas the random intercept for outgroup contact was negatively correlated with the random intercept for perceived discrimination (*r* = *−*.22, *SE* = .03, *p* < .001; 99% CI [−.31, −.16]). The correlation coefficients indicate small to medium associations between contact and perceived discrimination. These results show that those who reported greater ingroup contact across the four-wave assessment period also reported *higher* perceived discrimination across the same period, but those who reported greater outgroup contact across the four-wave assessment period reported *lower* perceived discrimination across the same period. This indicates that stable individual differences in contact and perceived discrimination correlate in the population but does not indicate a longitudinal effect in which changes in contact precede changes in perceived discrimination. The random intercept for ingroup contact was also positively correlated with the random intercept of outgroup contact (*r* = .27, *SE* = .02, *p* < .001; 99% CI [.21, .31]).

#### Within-Person Coefficients

When adjusting for between-person stability, the within-person effects of ingroup and outgroup contact on subsequent levels of perceived discrimination were nonsignificant, as were the within-person effects of perceived discrimination on subsequent levels of ingroup and outgroup contact (see Table 1, for all within-person effects in the model). This indicates that an individual’s deviation from their trait level of contact in 1 year was not associated with a deviation from their trait-level perceived discrimination 1 year later. The results also showed that the reciprocal association did not emerge. That is, an individual’s deviation from their trait level of perceived discrimination was not associated with a deviation from their trait-level contact 1 year later. These findings are not consistent with an effect of contact on perceived discrimination because they do not show that changes in contact precede changes in perceived discrimination or vice versa.^5^

Thus, the findings of Study 1 do not provide support for our hypotheses that ingroup contact predicts increased perceived discrimination over time or that outgroup contact predicts decreased perceived discrimination over time. Rather than a within-person effect of contact on perceived discrimination, our results showed time-invariant associations between contact and perceived discrimination that may reflect third-variable effects. We note that, in Study 1, we analyzed ordinal variables as if they were metric variables, which, as discussed in the next section, risks degrading statistical inferences (Liddell & Kruschke, 2018). We did so as the RI-CLPM has yet to be implemented or described for ordinal variables.

## Study 2

Study 1 investigated whether having more or less contact with ingroup and outgroup members predicts changes in disadvantaged-group members’ perceived discrimination and found no evidence of within-person effects. There are, however, other ways in which contact, especially friendships, may shape perceived discrimination. Through the process of socialization, disadvantaged-group members’ perceptions of discrimination might be influenced not merely by their amount of contact with ingroup and outgroup members but by those ingroup and outgroup members’ perceptions of discrimination. We explored this hypothesis in Study 2.

We focused specifically on friendships and used social network analysis to investigate socialization. Although individuals may be influenced by even unfamiliar others in short interactions (Blanchard et al., 1991), socialization is more likely to occur through long-term interactions with friends (Paluck, 2011). We used a sociometric measure of friendship, in which respondents self-report the specific individuals whom they consider as friends in their social network. This enabled us to assess whether friends’ perceptions of discrimination predict individuals’ perceptions of discrimination: We determined the perceptions of discrimination held by an individual’s friends by first identifying the friends each individual nominated in the network and then directly assessing those friends’ self-reported perceived discrimination. In this way, Study 2 investigated the associations between individuals’ perceptions of discrimination, their numbers of ingroup and outgroup friends, and their friends’ perceptions of discrimination.

### Method

#### Participants and Procedure

We recruited 1,445 high school students (*M*_age_ = 14.5; 718 girls, 664 boys, five other, 20 prefer not to say) from 10 schools across England as part of a larger study on intergroup contact among young people. Of these students, 692 (48%) identified as White, 466 (32%) as Asian, 161 (11%) as Black, 86 (6%) as mixed, and 40 (3%) identified as another ethnic group or did not respond to the relevant question.^6^ White students represent the advantaged majority group and Asian, Black, and mixed students represent disadvantaged minority groups. Because we were interested in perceived discrimination among members of disadvantaged minority groups, we included only Asian, Black, and mixed participants in the present study. Further, we had to exclude participants from one school in which too few students participated in the study. Accordingly, our final sample comprised 712 minority students (*M*_age_ = 14.5; 366 girls, 317 boys, 1 other, 7 prefer not to say) from nine schools, of whom 466 (65%) were Asian, 160 (22%) were Black, and 86 (12%) were mixed. In addition, we used responses from 666 White students as participants’ outgroup friends’ perceived discrimination when testing for socialization effects.

We tasked a survey company with collecting data from the same grade (Year 10, equivalent to 9th grade in the United States) at each school. Of all students enrolled in 9th grade, 72% participated in the study. We focused on the grade, rather than class, as the network boundary because students were allocated to different classes for different subjects and thus belonged to multiple cross-cutting class groups. Data were collected between January and March 2018. Students completed a pen-and-paper survey in a classroom session (40–45 min). Once completed, the surveys were sealed in an envelope and later coded and transcribed by the survey company. Surveys with an incomplete consent form were securely destroyed. The study was approved by the University Ethics Committee (R53809/RE002).

#### Measures

As part of a larger study, we included three measures relevant to the present study. The complete list of questions is available online (see online Appendix D).

##### Demographic Information

In two multiple-choice questions, participants reported their gender and ethnicity. We recoded participants’ self-reported ethnicities from the 19 possible choices to the four broader categories (White or White British, Asian or Asian British, Black or Black British, mixed or mixed British) that were used as reference groups in subsequent questions.

##### Friendship Nominations

**Figure 2 fig2:**
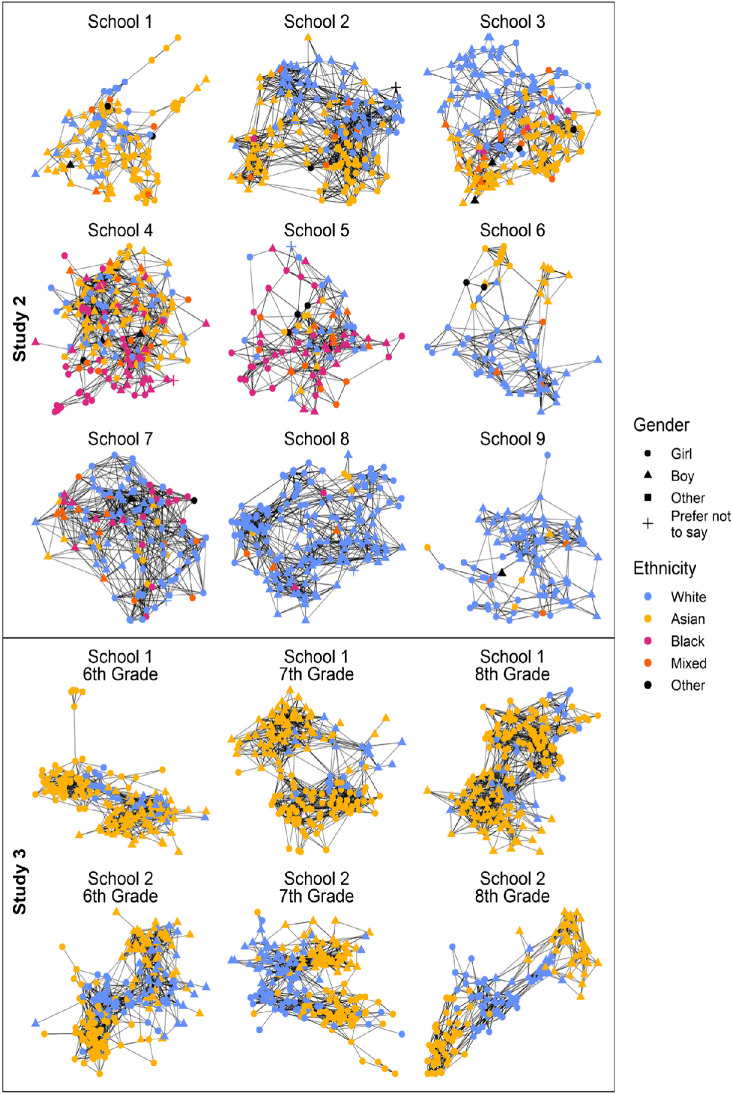
Network Graphs for Each School (Studies 2 and 3) *Note*. Each node represents a student, and each tie (link between nodes) represents a friendship. The figure shows each friendship network at Wave 1, with isolates removed. The networks are visualized using the ggraph R package (Pedersen, 2021), and the coordinates of each node in the network plot are determined using the force-directed Fruchterman–Reingold layout algorithm. See the online article for the color version of this figure.

Friendship networks within each grade were elicited using peer nomination procedures. Participants nominated up to 10 friends from their grade in response to the question, “Who are your best friends in Year 10 at your school?” Previous studies have used similar friendship measures capped at 10 nominations (e.g., Hjerm et al., 2018; Wölfer et al., 2017). The friendship networks for the nine schools are shown in Figure 2. The networks ranged in size from *n* = 75 to *n* = 236 students. Participants were instructed to write down the first and last name of each nominated friend and were reminded that their nominations were confidential. Coders at the survey company transcribed nominations and matched names to the lists of students provided by the schools. We received the matched dataset with all names replaced by anonymized identifiers. We used participants’ self-reported ethnicity, divided into the four broader categories, to calculate the numbers of ingroup and outgroup friends each participant had nominated.[Fig fig2]

##### Perceived Group Discrimination

Participants rated the extent to which they agreed or disagreed that “in our society today, there is a lot of discrimination or unfair treatment against” each of four groups: White or White British people, Asian or Asian British people, Black or Black British people, and mixed or mixed British people (1 = *strongly disagree*, 5 = *strongly agree*). As we were interested in perceived discrimination against the ingroup, we used participants’ rating for their own ethnic group (see the Demographic Information section) as the outcome variable in all analyses.

#### Analysis Strategy

We measured perceived discrimination, the outcome variable, using one ordinal item with five response options. Analyzing ordinal data as if they were metric data risks distorting estimates of effect sizes and inflating rates of false-positive and false-negative findings (Liddell & Kruschke, 2018). Accordingly, we used cumulative ordinal regression models, which estimated, for each observed response, how likely it was that a participant would choose each of the five ordered response options (see Bürkner & Vuorre, 2019, for an introduction). In these models, a positive regression coefficient implies that the log odds of a participant choosing a higher response option increase with each additional unit of the predictor variable. We used two kinds of models to test our hypotheses.

In Model 1, we estimated perceived discrimination against the participant’s ingroup as a function of the number of ingroup and outgroup friends the participant had. We used monotonic effects (Bürkner & Charpentier, 2020) to model the two ordinal predictor variables. That is, we estimated both the magnitude and direction of the average change for each additional friend and the proportion of the total change that occurs with each of the 10 possible friendship nominations. Using monotonic effects to model the number of ingroup and outgroup friends reflects the assumption that, for example, having one instead of no friends might have a greater effect than having 10 instead of nine friends.

In Model 2, we estimated perceived discrimination against the participant’s ingroup as a function of both the number of ingroup and outgroup friends the participant had and the participant’s ingroup and outgroup friends’ perceived discrimination against the participant’s ingroup. To do so, we used network autocorrelation to estimate ingroup and outgroup socialization effects (Leenders, 2002). For each participant, we calculated the average ratings of perceived discrimination against their ingroup from their ingroup friends and their outgroup friends. We *z*-standardized the average ratings by ingroup and outgroup friends across participants. We included these average ratings in the model as monotonic interactions with the number of ingroup and outgroup friends. The resulting regression coefficients estimated how much lower or higher the effect of each additional friend was for each additional standard deviation of the participant’s friends’ average perceived discrimination ratings. In both models, we included varying (random) intercepts to estimate differences in perceived discrimination across schools.

To estimate those models, we used the *brms* R package (Bürkner, 2017, 2018) as an interface to fit Bayesian generalized linear multilevel models in Stan (Stan Development Team, 2021). We used Bayesian statistical methods because monotonic effects, which were developed by Bürkner and Charpentier (2020), have, to our knowledge, only been implemented using Bayesian methods.^7^ Bayesian inference involves choosing a likelihood function and prior distributions. The likelihood function links the observed data to one or more model parameters (e.g., regression coefficients) by expressing how likely the observed data would have been for different values of the model parameters. Prior distributions state how plausible different values of the model parameters are before considering the observed data. Bayesian inference applies Bayes’ theorem to update prior distributions in light of the observed data to produce posterior distributions. These posterior distributions state how plausible different values of the model parameters are given the observed data. Our models derived the likelihood of the observed responses from a generalized linear model with a cumulative logit link function. Our models assigned weakly informative prior distributions to model parameters.^8^ The data, materials, and analysis code for Study 2 are provided in online Appendix D.

### Results and Discussion

Participants nominated, on average, four friends (*Mdn* = 4, *M* = 4.20, *SD* = 2.81). As to be expected (McPherson et al., 2001), participants nominated, on average, more friends from the disadvantaged ingroup (*Mdn* = 3, *M* = 3.06, *SD* = 2.62) than from the advantaged outgroup (*Mdn* = 0, *M* = 1.14, *SD* = 1.89; Cohen’s *d* = 0.84). Likewise, participants’ ingroup friends perceived, on average, more discrimination against the participants’ ingroup (*M* = 4.04, *SD* = 0.63) than their outgroup friends did (*M* = 3.44, *SD* = 0.75; Cohen’s *d* = 0.87). Descriptive statistics are presented in online Appendix B.

**Figure 3:**
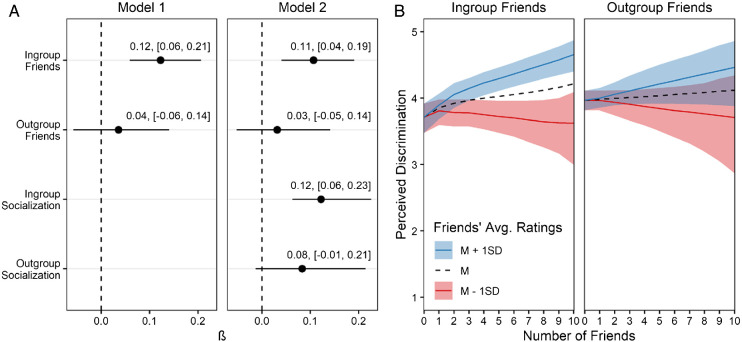
Results From Study 2 *Note*. (A) Coefficients (with 95% uncertainty intervals) estimating the average change in perceived discrimination (in log odds) for each additional friend a participant had and by how much that average change was greater for each additional standard deviation in the friends’ average rating of perceived discrimination against the participant’s ingroup. (B) Predictions (with 95% uncertainty intervals) from Model 2 show the interaction implied by the ingroup and outgroup socialization effects. We used the median number of outgroup friends when plotting the effect of the number of ingroup friends and vice versa. See the online article for the color version of this figure.

Figure 3 shows the results of our focal analyses. We found that the number of ingroup friends predicted greater perceived discrimination in both Model 1 (β = 0.12, [0.06, 0.21]) and Model 2 (β = 0.11, [0.04, 0.19]). These results support our hypothesis that greater ingroup contact predicts increased perceived discrimination. Furthermore, Model 2 revealed that the participant’s ingroup friends’ average ratings of perceived discrimination moderated the effect of the number of ingroup friends (β = 0.12, [0.06, 0.23]): The greater the ingroup friends’ perceived discrimination, the greater the individual’s perceived discrimination. This result is in line with our hypothesis that ingroup friends’ perceptions of discrimination influence individuals’ perceptions of discrimination. In contrast, we found no evidence for the corresponding effect of the participants’ outgroup friends’ average ratings of perceived discrimination against the participant’s ingroup (β = 0.08, [−0.01, 0.21]), which aligns with our expectation that socialization occurs via relationships with ingroup, but not outgroup, members. Finally, we found that the number of outgroup friends did not predict perceived discrimination in Model 1 (β = 0.04, [−0.06, 0.14]) or Model 2 (β = 0.03, [−0.05, 0.14]). Thus, the findings did not support our hypothesis that greater outgroup contact predicts decreased perceived discrimination. In summary, Study 2 provided evidence that ingroup, but not outgroup, friendship is associated with perceived discrimination.^9^

To put these effects into perspective, our results implied that a student who has three ingroup friends will perceive, on average, more discrimination against the disadvantaged ingroup (*M* = 3.97, [3.82, 4.11]) than a student who has no ingroup friends (*M* = 3.72, [3.48, 3.92]; Cohen’s *d* = 0.27, [0.06, 0.49]).^10^ Consistent with the hypothesized socialization effect, our results indicate that this difference would be greater if the student’s three ingroup friends had reported above average (+1 *SD*) perceived discrimination (Cohen’s *d* = 0.45, [0.23, 0.70]) and smaller if they had reported below average (–1 *SD*) perceived discrimination (Cohen’s *d* = 0.07, [–0.19, 0.34]).

## Study 3

To estimate socialization effects, Study 2 used network autocorrelation, which is a common approach to modeling social influence in cross-sectional social network data (Leenders, 2002). In this sense, the findings from Study 2 provided evidence that ingroup, but not outgroup, socialization is associated with perceived discrimination in disadvantaged groups. Without longitudinal social network data, however, we cannot rule out that selection effects account for the observed association. We would find evidence for network autocorrelation if, as hypothesized, participants had become more similar to their friends over time, but also if participants had chosen to form friendships with people who share similar perceptions of discrimination. To distinguish these explanations, we need to consider longitudinal social network dynamics. Study 3 used longitudinal social network analysis to examine the direction of the associations identified in Study 2. Specifically, by employing a five-wave design, Study 3 investigated the extent to which the similarity in perceived discrimination among ingroup friends resulted from friends’ perceptions becoming more similar over time (i.e., socialization) or individuals choosing friends with similar perceptions (i.e., selection).

### Method

#### Participants and Procedure

We utilize data from a longitudinal social network study conducted in two schools in a town in North West England (Bracegirdle et al., 2022). The town had a population of 77% White and 19% Asian residents (Office for National Statistics, 2011) and sizeable numbers of students from the White and Asian communities attended the two schools (School 1: 16% White, 82% Asian; School 2: 39% White, 55% Asian).

All students in 6th (aged 11–12), 7th (aged 12–13), and 8th (aged 13–14) grades were invited to participate in the study. Of the 1,445 students enrolled across the three grades, 1,328 (92%) participated. Because our measures were tailored to Asian and White students, we excluded 158 students who did not report their ethnicity (*n* = 113), reported different ethnicities across waves (*n* = 4), or reported an ethnicity other than Asian or White (*n* = 40 Black/Black British; *n* = 1 Chinese/Chinese British). This left a final sample of 1,170 students (829 Asian, 341 White; 612 girls, 558 boys; *M*_age_ = 12.11, *SD* = 0.89).

Five waves of data were collected over the academic year 2017/2018, with 6- to 8-week intervals between waves. Of the final sample, 84% participated in Wave 1, 81% in Wave 2, 80% in Wave 3, 79% in Wave 4, and 77% in Wave 5. Dropout at each wave was, at most, weakly correlated (mean absolute |*r*| = .06) with participant attributes measured at the preceding wave.

At each wave, students completed a pen-and-paper survey in a classroom session (30–50 min). A team of researchers visited each classroom to explain the study and answer any questions. Each survey contained the measures, an information sheet, and an informed consent form. Additionally, parents of all students received information sheets and opt out consent forms 2 weeks before the study. Less than 1% of parents indicated that they did not want their child to participate. The study was approved by the University Ethics Committee (R52944/RE001).

#### Measures

Here, we report measures relevant to our hypotheses. The full questionnaire is available online (see online Appendix D). All measures were included at each wave.

##### Demographic Information

The schools provided information about students’ age and gender, while students reported their own ethnicity. Students were asked, “Which of the following ethnic groups do you think best describes you?” and responded by selecting one of the 10 options. The sample included only students who selected either Asian or White.

##### Friendship Nominations

Students nominated up to 10 friends in response to the question, “Who are your friends (in your year group [i.e., grade])?” Accordingly, we examined six friendship networks: Grades 6, 7, and 8 at each of the two schools. The six friendship networks are shown in Figure 2. The networks ranged in size from *n* = 163 to *n* = 221 students. Ingroup and outgroup friendships were identified based on the correspondence between individuals’ self-reported ethnicity and their friends’ self-reported ethnicity. Less than 1% of friendship nominations were unmatchable. For details, see online Appendix C.

##### Perceived Group Discrimination

Students reported the extent to which they agreed or disagreed with the statement, “There is a lot of unfair treatment towards [INGROUP] people in Britain today” (1 = *disagree strongly*, 5 = *agree strongly*). “Ingroup” was replaced with “Asian” or “White” depending on each student’s ethnicity. Thus, the target of discrimination differed across students from the two ethnic groups. Accordingly, Studies 2 and 3 considered alternative types of outgroup socialization. Study 2 assessed whether outgroup friends’ perceptions of discrimination against the participant’s ingroup predict participants’ perceived discrimination against their ingroup (e.g., whether White friends’ perceptions of discrimination against Asian people predict Asian students’ perceptions of discrimination against Asian people). Study 3 considered whether outgroup friends’ perceptions of discrimination against their own ingroup predict participants’ perceived discrimination against their ingroup (e.g., whether White friends’ perceptions of discrimination against White people predict Asian students’ perceptions of discrimination against Asian people).

#### Analysis Strategy

We examined the coevolution of friendship networks and perceived discrimination using stochastic actor-oriented models (Snijders et al., 2010) implemented in RSiena (Ripley et al., 2019). RSiena coevolution models use simulation methods to determine how the network and individuals’ attributes (e.g., perceived discrimination) change over time, given specific effects that are predicted to influence change. The models consist of two types of components, termed “behavioral dynamics” and “network dynamics” (Steglich et al., 2006). In the behavioral dynamics, we modeled change over time in individuals’ perceived discrimination, and tested contact and socialization effects. The estimated effects can be considered as multinomial logistic regression coefficients for ordered dependent outcomes, indicating the extent to which each effect predicts changes in perceived discrimination. In the network dynamics, we modeled change over time in friendships, and tested selection effects. The estimated effects can be seen as categorical logistic regression coefficients for binary outcomes, indicating the extent to which each effect predicts changes in friendships. The simultaneous estimation of the behavioral and network dynamics enabled us to investigate whether friendships longitudinally predict changes in perceived discrimination and whether perceived discrimination longitudinally predicts changes in friendships. Thus, we could test the extent to which the similarity in perceived discrimination among friends results from friends influencing each other over time (i.e., socialization) or individuals choosing to befriend similar others (i.e., selection).

In the following subsections, we summarize the effects included in the behavioral and network dynamics. As the present research examines perceived discrimination among disadvantaged ethnic groups, we estimated separate effects for Asian and White students and present the results for Asian students. To separate group-dependent contact effects and socialization effects, we used the modeling strategy developed by Bracegirdle et al. (2022). Full details of our analyses are provided in online Appendix C. We provide only a brief outline of RSiena coevolution modeling specifically applied to the present study and refer readers to prior work for a more detailed overview (e.g., Snijders et al., 2007; Steglich et al., 2006).

##### Behavioral Dynamics

We included two contact effects: the effects of number of ingroup (outgroup) friends on perceived discrimination, which tested whether individuals with a greater number of ingroup (outgroup) friends reported higher or lower perceived discrimination over time. Next, we included two socialization effects: The effects of ingroup (outgroup) friends’ perceived discrimination on individuals’ perceived discrimination, which tested whether individuals’ perceptions of discrimination became more similar to their ingroup (outgroup) friends’ perceptions of discrimination over time. We also included four controls: the linear and quadratic shape effects (which reflect overall changes in perceived discrimination with time) and the main effects of ethnicity and gender.

##### Network Dynamics

We controlled for confounding friendship selection processes in the network dynamics. We included four selection effects that controlled for the potential influence of perceived discrimination on friendship choices. These consisted of ego perceived discrimination effects, which capture the influence of individuals’ perceptions of discrimination on the number of friendship nominations sent to ingroup and outgroup peers, and perceived discrimination similarity effects, which capture individuals’ tendencies to befriend ingroup and outgroup peers with similar perceptions of discrimination to themselves. Next, we included six effects controlling for the influence of demographic variables on network structure: We controlled for students’ own (ego effect) and their friends’ (alter effect) ethnicity and gender, and students’ homophilous preferences for same-ethnic and same-gender friends. Finally, we controlled for 12 structural effects, which capture how the network itself influences friendship choices.^11^

##### Modeling Approach

We estimated two RSiena coevolution models. Model 1 examined contact effects on perceived discrimination, in line with previous research. Model 2 included all the effects estimated in Model 1, but also estimated socialization effects. In each model, the grade networks were combined using the RSiena multigroup option. The multigroup approach accounted for the nested data structure and resulted in a larger sample, which provided sufficient statistical power to estimate the complex model specification (Stadtfeld et al., 2020). Missing data were treated using model-based imputation (Huisman & Steglich, 2008; Zandberg & Huisman, 2019). To determine the reliability of our results, we assessed convergence and goodness-of-fit. The results indicated that both models showed good convergence and adequately fitted the data (see online Appendix C). The data, materials, and analysis code for Study 3 are provided in online Appendix D.

### Results and Discussion

On average, students nominated seven friends (*M* = 7.12, *SD* = 2.03). Both Asian and White students nominated more ingroup friends (*M* = 6.15, *SD* = 2.29) than outgroup friends (*M* = 0.97, *SD* = 1.29; Cohen’s *d* = 1.66). This indicates a high level of ethnic segregation in the friendship networks, as shown above in Figure 2. Consistent with expectations, Asian students perceived greater ingroup discrimination (*M* = 3.46, *SD* = 1.05) than White students (*M* = 3.12, *SD* = 1.05; Cohen’s *d* > 0.22). The networks showed sufﬁcient change over time to model the coevolution of friendships and perceived discrimination. The Jaccard index (which calculates stability in the network as the similarity of friendship ties between waves) ranged from .34 to .52, which indicates a suitable balance between network stability and change for RSiena coevolution modeling (Ripley et al., 2019). Descriptive statistics are shown in online Appendix C.

Table 2 shows the results for the two RSiena coevolution models. The estimates (log-odds ratios) in the behavioral dynamics represent the likelihood of changes in an individual’s perceived discrimination based on each respective effect, while the estimates (log-odds ratios) in the network dynamics represent the likelihood that an individual will form or maintain a friendship tie based on each respective effect.[Table tbl2]

#### Behavioral Dynamics

We tested contact effects in both models. We hypothesized that having more ingroup friends would predict *increased* perceived discrimination over time, whereas having more outgroup friends would predict *decreased* perceived discrimination over time. The results did not support these hypotheses: neither students’ number of ingroup friends, nor students’ number of outgroup friends, significantly predicted changes in perceived discrimination (as indicated by the nonsignificant estimates for number of ingroup friends and number of outgroup friends). Thus, in contrast to prior research, yet consistent with Study 1, we found that ingroup and outgroup contact do not predict changes in perceived discrimination among ethnic minority students.

We tested socialization effects in Model 2. We hypothesized that ingroup friends’ perceptions of discrimination would influence individuals’ perceptions of discrimination, such that their perceptions become more similar over time. The results supported this hypothesis: Students’ perceptions of discrimination became more similar to their ingroup friends’ perceptions of discrimination over time (as indicated by the positive estimate for ingroup friends’ perceived discrimination). These findings suggest that socialization explains how ingroup friendships shape individuals’ perceptions of discrimination. Importantly, we identified this effect while controlling for the possible tendency to select ingroup friends with similar perceptions of discrimination.

#### Network Dynamics

We controlled for the potential effects of perceived discrimination on friendship choices (selection effects). We found, however, that neither individuals’ nor their friends’ perceptions of discrimination predicted friendship selection (as indicated by the nonsignificant estimates for Ego Perceived Discrimination × Ingroup/Outgroup Friends and ingroup/outgroup friends’ perceived discrimination similarity). These findings suggest that perceived discrimination is not an attribute that influences students’ friendship choices. Most importantly, students did not prefer to befriend ingroup peers with similar perceptions of discrimination to themselves. Accordingly, the results of the RSiena coevolution models indicate that the similarity in perceived discrimination among ingroup friends occurs because individuals are influenced by their ingroup friends’ perceptions of discrimination (i.e., socialization) and not because individuals select ingroup friends with similar perceptions of discrimination (i.e., selection). Study 3 thus extends the results from Study 2 by indicating the direction of the association between individuals’ perceived discrimination and their ingroup friends’ perceived discrimination. Additionally, we found that students were more likely to form and maintain friendships with ingroup members than with outgroup members (as indicated by the positive estimate for same ethnicity), which may provide more opportunities for ingroup socialization to occur over time.^12^

## General Discussion

The present research combined longitudinal and social network data with state-of-the-art statistical models to investigate how social relationships—with ingroup *and* outgroup members—may shape perceived discrimination in disadvantaged groups. First, we tested the hypotheses that contact with an advantaged outgroup predicts *less* perceived discrimination, while contact with the disadvantaged ingroup predicts *more* perceived discrimination. Study 1 was the first longitudinal study that included both outgroup and ingroup contact. The results showed that, on average, individuals who are higher on outgroup contact are lower on perceived discrimination, whereas those who are higher on ingroup contact are higher on perceived discrimination. The results did not, however, provide support for a within-person effect whereby changes in an individual’s contact *precede* changes in their perceived discrimination.

Second, we tested the hypothesis that disadvantaged-group members’ perceptions of discrimination would be predicted not merely by how many ingroup and outgroup friends they had (*contact effects*), but by those friends’ perceptions of discrimination (*socialization effects*). Study 2 provided cross-sectional evidence for both ingroup contact and ingroup socialization: Having ingroup friends was associated with greater perceived discrimination, and the association was stronger when those ingroup friends perceived more discrimination against the disadvantaged ingroup. Study 3 used longitudinal social network data to show that ingroup friends have similar perceptions of discrimination because ingroup friends’ perceptions become more similar over time (i.e., socialization), not because individuals befriend those with similar perceptions (i.e., selection). Together, these studies suggest that disadvantaged-group members look to their ingroup friends, not their outgroup friends, to inform their beliefs about discrimination. In the following sections, we discuss how our findings qualify and expand the current understanding of perceived discrimination and consider the strengths and limitations of our studies.

### Implications

Most research investigating how social relationships shape disadvantaged-group members’ perceptions of discrimination has focused on contact with the advantaged outgroup. Initial evidence suggested that such outgroup contact is associated with less perceived discrimination (Dixon et al., 2010; Sengupta & Sibley, 2013; Tropp et al., 2012). In contrast, we found no evidence, across three studies, that outgroup contact predicts reduced perceived discrimination in disadvantaged minority groups. On the one hand, our findings are consistent with a recent meta-analysis showing that the associations between outgroup contact and perceived discrimination are small and variable across studies (Reimer & Sengupta, 2023). In that sense, it is perhaps unsurprising that we did not find evidence for negative associations between intergroup contact and perceived discrimination. On the other hand, Studies 1 and 3 are two of only five studies examining this relationship longitudinally (Koschate et al., 2012; Reimer et al., 2017; Tropp et al., 2012) of whom only Tropp et al. (2012) found evidence for the hypothesized negative association between outgroup contact and perceived discrimination. Further, we provide the only longitudinal study that controlled for ingroup contact and did not conflate within-person change and between-person stability (Hamaker et al., 2015). Thus, the present research casts doubt on the hypothesis that contact with the advantaged impacts perceived discrimination among the disadvantaged.

Instead, the present research highlights the important role of social relationships with *ingroup* members in shaping disadvantaged-group members’ perceptions of discrimination. Our findings indicate that perceived discrimination is shaped through the process of socialization—disadvantaged-group members’ perceptions of discrimination became more similar to their ingroup friends’ perceptions of discrimination over time. The present research thus provides initial evidence for the claim made in earlier research that socialization is the mechanism by which ingroup contact shapes perceived discrimination (Levin et al., 2006; Sengupta, Milojev, et al., 2015; Sengupta & Sibley, 2013). Furthermore, we found that socialization occurred only through relationships with ingroup, not outgroup, members. This finding advances recent research revealing the importance of the ingroup for socialization (Bracegirdle et al., 2022) and supports broader theoretical and empirical work positing that individuals are influenced primarily by ingroup norms and members (Abrams et al., 1990; Sherif & Sherif, 1953; Turner et al., 1987). Additionally, socialization may have occurred through relationships with ingroup, not outgroup, members because group discrimination may be more frequently discussed among members of disadvantaged groups (Saguy et al., 2008; Sanchez et al., 2022).

Our study also adds new evidence concerning prior theorizing on shared reality and the relational nature of political attitudes. In a seminal article, Jost et al. (2003) argued that people gravitate toward sociopolitical beliefs that help fulfill unmet psychological needs. Jost et al. (2009) refined this argument and identified three needs that could be fulfilled by political attitudes: the existential need for security, epistemic need for control, and relational need to belong. Of these, relational needs remain the most underresearched (Osborne et al., 2019; but see Bahamondes, Sengupta, et al., 2021). The theorized importance of relational needs is based on shared reality theory (Hardin & Higgins, 1996), which posits that people strive for a shared understanding of their social world and so adjust their attitudes in the direction of significant others (Jost et al., 2008). Our study presents evidence of shared reality processes operating in the political domain, as the results indicate that members of disadvantaged groups arrive at a common understanding of the level of discrimination faced by their group in society through ingroup socialization. By extension, our study may be considered to provide evidence that relational needs shape political attitudes, although ingroup socialization may also serve existential or epistemic needs. The links between ingroup socialization and psychological needs remain an open area for further research that measures relational, existential, and epistemic needs. Future research should also investigate the role of ingroup socialization in fostering other types of political attitudes among both disadvantaged and advantaged groups (e.g., collective narcissism; Cichocka, 2016). Furthermore, future studies should investigate whether socialization occurs only through ingroup friends or also through contact with other ingroup members.

The present research was only able to reveal the importance of socialization for perceived discrimination by using social network analysis. Prior research has postulated the role of socialization in shaping perceived discrimination, yet could not test this process directly. This is because prior work could only account for contact with ingroup and outgroup members, and not the levels of perceived discrimination among those ingroup and outgroup members. As such, previous research has considered only contact, not socialization, effects. The present research applied recent methodological advances in social network analysis (Bracegirdle et al., 2022) to separate contact and socialization effects for both ingroup and outgroup friends. Our findings demonstrate the necessity of using social network methodology to capture socialization when investigating how social relationships influence perceived discrimination. Social network analysis remains an underused approach in social psychology (Wölfer & Hewstone, 2017), and the distinction between contact and socialization effects is seldom considered in social–psychological research despite being well established in other areas, such as developmental psychology (Bukowski et al., 1998; Hartup, 1996). Thus, we hope our findings inspire future social–psychological research to apply our network analytic approach and disentangle contact and socialization processes when studying perceived discrimination and other sociopolitical beliefs and ideologies.

### Limitations

Across three studies, we conducted a rigorous investigation of social relationships and perceived discrimination in disadvantaged groups. Still, there are several limitations that qualify the extent to which our findings permit causal inferences and generalize to other contexts and age groups.

Our findings are based on research conducted in specific cultural contexts. On the one hand, our samples included Māori, Pacific Islander, and Asian respondents in New Zealand, as well as Black, Asian, and mixed respondents in the United Kingdom. Accordingly, we provided insights into the psychology of a diverse set of ethnic groups in two countries. On the other hand, the generalizability of our findings is limited in that the two countries we studied are English-speaking minority-world countries that do not represent the breadth of human values and experiences (Henrich et al., 2010). There is some evidence that the effects of contact on perceived discrimination might vary across societal contexts. For example, country-level analyses provided tentative evidence that the association between outgroup contact and perceived discrimination might be larger in countries with greater cultural distance from the United States (Reimer & Sengupta, 2023). In order to determine the extent to which the present findings generalize to other cultural contexts, future research should examine how ingroup and outgroup contact shape perceived discrimination in contexts beyond the minority-world societies that dominate psychological research.

Other contextual factors may have also influenced our findings. Our hypotheses relate to the contact disadvantaged groups have with their own group and with advantaged groups. The distinction between disadvantaged and advantaged groups is based on differences in resources, power, and social status, and often, but not always, aligns with differences in numerical status. In both New Zealand and the United Kingdom, the disadvantaged ethnic groups that we sampled are numerical minority groups in society. In Study 3, however, Asian students are the numerical majority group in the school context. The different intergroup dynamics in the local and broader social context may influence perceptions of discrimination and contact and socialization processes. For example, Asian students, as the numerical majority group, will have more opportunities for ingroup contact than outgroup contact in the school environment, which could increase the prevalence of ingroup socialization. Future research should seek to explore potential differences in the socialization of perceived discrimination depending on numerical majority–minority status.

Further characteristics of the present research may have influenced our findings regarding outgroup contact. We examined positive outgroup contact and friendship and found that neither predicted changes in perceived discrimination. This does not preclude the possibility that other forms of outgroup contact influence perceived discrimination. Individuals can experience intergroup contact in many forms that extend beyond positive contact or friendship, such as negative contact, indirect contact, or incidental but regular neutral contact with outgroup members in the school or work environment. These alternative forms of contact, which were not assessed in the present research, may shape individuals’ perceptions of discrimination. For example, negative contact experiences may be associated with increased perceived personal discrimination and, in turn, perceived group discrimination (Reimer et al., 2017). Accordingly, by focusing only on positive contact and friendship, the present research may have overlooked other relevant contact experiences.

Due to the time lags employed in the present research, we examined whether outgroup contact longitudinally predicted perceived discrimination 2 months to 1 year later. Prior longitudinal research on outgroup contact and perceived discrimination has employed similar time lags, ranging from 6 months to 2 years (Koschate et al., 2012; Reimer et al., 2017; Tropp et al., 2012). It is unusual for a longitudinal test of the contact hypothesis to allow for a comparison between two different lags, as our study does, allowing for both shorter acting or longer lasting contact effects to emerge. Nonetheless, it remains possible that the lags in our studies were either too short for the effects to emerge or too long for them to endure. In the absence of any prior theorizing on the expected timespan for contact effects, this remains an open question. Future research should seek to identify the ideal time lag between assessments and subsequently test whether the present findings hold across contexts and contact forms.

We investigated contact effects among both adults (Study 1) and adolescents (Studies 2 and 3). We focused on adolescence because it is a key period in the development of sociopolitical attitudes and ethnic identity (Jugert et al., 2020; Krosnick & Alwin, 1989), making it crucial for understanding how perceptions of ethnic discrimination form. However, recent meta-analytic evidence, albeit exploratory, suggests that the negative association between outgroup contact and perceived discrimination might be weaker among adolescents than adults (Reimer & Sengupta, 2023). It is, therefore, perhaps unsurprising that we did not find negative associations between intergroup contact and perceived discrimination in our adolescent samples. Yet, we also found no evidence that outgroup contact predicts reduced perceived discrimination in our random sample of ethnic minority adults.

While we investigated contact effects among both adults and adolescents, we only examined socialization effects among adolescents (Studies 2 and 3). It is plausible that socialization effects are more pronounced among adolescents who tend to be more susceptible than adults to social inﬂuence (Telzer et al., 2018). Therefore, our findings do not provide conclusive evidence that socialization also explains how, if at all, ingroup friendships shape perceived discrimination in early adulthood and beyond. Social network studies of socialization tend to focus on adolescents because schools provide relevant and bounded network structures. Still, future studies should investigate the socialization of perceived discrimination in universities, companies, and other bounded organizations to determine whether the present findings replicate across the life span.

Even though our findings are based on nonexperimental studies, we used sophisticated longitudinal and social network analysis methods to examine the direction of the observed associations between friendships and perceived discrimination. In contrast to earlier longitudinal research (e.g., Reimer et al., 2017), we used a RI-CLPM (Hamaker et al., 2015) to disentangle between-person stability and within-person change in contact effects. While estimating within-person change is not sufficient to infer a causal effect (Rohrer & Murayama, 2021), it satisfies some of the assumptions for causal inference by ruling out time-invariant confounders (e.g., stable individual differences in personality or ideology). Furthermore, we used longitudinal social network analysis to disentangle contact, socialization, selection, and other network effects (Ripley et al., 2019). In so doing, we rule out selection as an alternative explanation: Although our results indicate that adolescents tended to preferentially befriend same-gender and same-ethnicity peers, there was no evidence that they preferentially befriended peers with similar perceptions of discrimination. These results corroborate research showing that, when choosing friends, similarity in sociodemographic characteristics is more important than similarity in other attributes (McPherson et al., 2001). By separating between- and within-person effects and disentangling contact, socialization, and selection processes, the present research advances our understanding of the direction of the associations between friendships and perceived discrimination.

Nevertheless, our analyses could not provide definitive evidence of causal effects because, without random assignment, we cannot rule out that omitted time-variant variables confounded what we identiﬁed as the effects of contact and socialization (Lomi et al., 2011). For example, in Study 3, it is conceivable that adolescents who became friends shared an environment that exposed them to experiences that, in turn, caused their perceptions of discrimination to become more similar to each other. In principle, experimental research could estimate the causal effects of ingroup and outgroup friendship on perceived discrimination. In practice, however, it is extremely difficult for researchers to manipulate the quantity and quality of people’s social relationships. Experimental studies tend to create short-term “intergroup interactions” rather than long-term “intergroup contact” (MacInnis & Page-Gould, 2015) that are quite distinct from the intimate friendships we hypothesize to shape perceptions of discrimination. Instead, future research should look to natural and field experiments (e.g., Shook & Fazio, 2008) to establish the causal effects of friendships in naturalistic settings.

### Conclusion

The present research tested the proposition that subjective perceptions of group discrimination are shaped by social contact and social influence processes. We used state-of-the-art longitudinal and social network analyses to investigate whether having ingroup and outgroup friends, as well as those ingroup and outgroup friends’ perceptions of discrimination, longitudinally predict disadvantaged-group members’ perceptions of discrimination while controlling for friendship selection processes. Across three studies, our results indicate that members of disadvantaged minority groups look to their ingroup friends, and not their outgroup friends, to inform their perceptions of discrimination. We further present initial evidence that socialization provides the mechanism by which ingroup friendships influence perceived discrimination. In so doing, the present research lays the groundwork for a comprehensive theoretical account of how social relationships shape perceptions of discrimination in disadvantaged groups.

## Figures and Tables

**Table 1:** Parameter Estimates for the Within-Person Effects of Every Variable at Time t − 1 on Each Variable at Time t

Time *t*	Time *t* − 1	*b*	*SE*	*z*	[99% CI]	
					*LL*	*UL*
Ingroup contact	Ingroup contact	0.04	0.03	1.46	−0.03	0.09
	Outgroup contact	0.02	0.02	2.18	−0.02	0.09
	Perceived discrimination	−0.01	0.02	−0.28	−0.05	0.03
Outgroup contact	Ingroup contact	0.02	0.02	1.19	−0.03	0.06
	Outgroup contact	0.06	0.03	2.18	−0.02	0.11
	Perceived discrimination	0.02	0.02	0.93	−0.03	0.05
Perceived discrimination	Ingroup contact	0.01	0.03	0.27	−0.07	0.07
	Outgroup contact	−0.04	0.03	−1.10	−0.12	0.03
	Perceived discrimination	< 0.01	0.03	0.01	−0.07	0.06
*Note*. *SE* = standard error; CI = confidence interval; *LL* = lower limit; *UL* = upper limit.						

**Table 2 tbl2:** Multigroup RSiena Coevolution Models

Effect	Model 1			Model 2		
	Est.	*SE*	*p*	Est.	*SE*	*p*
Behavioral dynamics						
Asian students						
Number of ingroup friends	0.01	0.02	.838	−0.01	0.02	.761
Number of outgroup friends	−0.05	0.03	.123	−0.04	0.03	.205
Ingroup friends’ perceived discrimination				0.16*	0.07	.026
Outgroup friends’ perceived discrimination				0.22	0.64	.732
Controls						
Linear shape	0.20*	0.10	.049	0.27*	0.10	.009
Quadratic shape	−0.17*	0.02	<.001	−0.07	0.05	.112
Ethnicity	−0.09	0.22	.696	−0.16	0.24	.506
Gender	−0.02	0.04	.662	−0.01	0.04	.947
Network dynamics						
Asian students						
Ego Perceived Discrimination × Ingroup Friends	−0.01	0.01	.348	−0.01	0.01	.332
Ego Perceived Discrimination × Outgroup Friends	−0.03	0.03	.231	−0.03	0.03	.279
Ingroup friends’ perceived discrimination similarity				0.02	0.08	.788
Outgroup friends’ perceived discrimination similarity				0.14	0.22	.534
Controls						
Alter ethnicity	−0.13*	0.02	<.001	−0.13*	0.02	<.001
Ego ethnicity	0.12*	0.02	<.001	0.11*	0.02	<.001
Same ethnicity	0.24*	0.02	<.001	0.24*	0.02	<.001
Alter gender	−0.01	0.02	.861	−0.01	0.02	.877
Ego gender	0.05*	0.02	.028	0.05*	0.02	.027
Same gender	0.60*	0.02	<.001	0.60*	0.02	<.001
*Note*. The behavioral dynamics models perceived discrimination and the network dynamics models friendship choices. Model 1 tests contact effects and the corresponding selection effects. Model 2 tests contact effects, socialization effects, and the corresponding selection effects. Models include the effects for structural controls and White students shown in online Appendix C. Est. = estimate; *SE* = standard error. *N* = 1,170. Ethnicity coded as 1 = White, 2 = Asian. Gender coded as 1 = boy, 2 = girl.						
* p < .05.						

## Footnotes

1 In addition, Carter et al. (2019) found that, in a diverse sample of U.S. college students, having friends from underrepresented minorities was associated with perceiving more discrimination against these minorities, while having White friends was associated with perceiving less discrimination. This study did not, however, differentiate the effects of contact on minority- and majority-group students.

2 The three studies relied on existing data and were not preregistered.

3 These patterns of missingness reflect the NZAVS booster sampling. For a more detailed analysis of missingness patterns, see online Appendix A.

4 We provide all supplemental material in the online Appendices on the Open Science Framework, available at https://osf.io/4n7vc.

5 The NZAVS contains two different measures of perceived discrimination and two different measures of contact. In the analysis reported here, we used the measures most similar to those employed in prior research. To verify that our findings replicate across the different measures, we conducted three robustness checks, which are reported in online Appendix A. The robustness checks using the alternative measures produced highly similar results to the main analysis, increasing confidence in our findings.

6 In the United Kingdom, “Asian” tends to refer to people of Indian, Pakistani, and Bangladeshi ethnicities (Goh & McCue, 2021), who make up 5.3% of the population of England and Wales (Office for National Statistics, 2018).

7 That said, the difference between frequentist and Bayesian approaches will not be all that consequential for most practical purposes and the present research. As we use weakly informative prior distributions, experience suggests that uncertainty intervals, based on Bayesian methods, and confidence intervals, based on frequentist maximum likelihood methods, will largely overlap.

8 Our models used conservative, weakly informative prior distributions for all fixed effects, β ∼ Student-*t*(3, 0, 2.5) on the log-odds scale, and for the proportion of the overall change with each additional friend, ζ ∼ Dirichlet(1). Our models used a Half-Student-*t*(3, 0, 2.5) prior for the standard deviation of the varying intercepts across schools. Our models used noninformative priors for the thresholds of the cumulative logit link function.

9 In online Appendix B, we report a replication of these analyses using a broader conceptualization of the ingroup. That is, we treated every friend who is a member of a disadvantaged group, but not necessarily of the participants’ own ethnic group, as an ingroup friend. The results provided support for our hypothesis regarding ingroup socialization (i.e., the greater the ingroup friends’ perceived discrimination, the greater the individual’s perceived discrimination), but, in line with the findings from Study 1, did not provide support for our hypotheses regarding ingroup and outgroup contact (i.e., the amount of ingroup and outgroup contact alone did not predict perceived discrimination).

10 Effect sizes are based on predictions from Model 2 and assume that, unless otherwise stated, a student has the median number of outgroup friends, and the students’ ingroup friends reported average perceived discrimination. Predictions do not account for school-level fixed intercepts. We focus on the effect of having an additional three ingroup friends, as three is both the median number of ingroup friends and that numbers’ median absolute deviation, a measure of dispersion that is a robust alternative to the standard deviation.

11 Structural effects capture endogenous network processes that influence friendships. For example, the reciprocity effect reflects individuals’ tendencies to reciprocate friendships. Structural effects represent important confounding effects; their exclusion from the model can lead to biases in the estimates for other effects, including those used in testing the hypotheses (Snijders et al., 2007). We included four fundamental structural effects recommended for all RSiena models (Ripley et al., 2019): “outdegree,” “reciprocity,” “gwespFF,” and “transitive reciprocated triplets.” In accordance with recommended practice, we included eight additional structural effects based on iterative goodness-of-fit testing (Lospinoso & Snijders, 2019): “gwespBB,” “three-cycles,” “indegree popularity,” “outdegree popularity,” “outdegree activity,” “reciprocal degree popularity,” “reciprocal degree activity,” and “truncated outdegree.” The inclusion versus exclusion of the additional structural effects in the model only impacted goodness-of-fit and did not impact the results relating to the hypotheses. For full details, including descriptions of the 12 structural effects, see online Appendix C.

12 To verify that our results were robust across alternative model specifications, we conducted three robustness checks. First, we reran the analyses including only fundamental structural effects (specified in Ripley et al., 2019) in the network dynamics. Second, we reran the analyses using an alternative specification of socialization (average similarity instead of total similarity). Finally, we reran the analyses including only Asian (not White) students in the model (which required a simpler model but precluded testing outgroup contact effects and introduced missing data). These three analyses are presented in online Appendix C. The robustness checks produced highly similar results to the main analysis. Specifically, all three robustness checks provided evidence of ingroup socialization effects, but neither selection nor outgroup contact effects.
